# Orbit image analysis machine learning software can be used for the histological quantification of acute ischemic stroke blood clots

**DOI:** 10.1371/journal.pone.0225841

**Published:** 2019-12-05

**Authors:** Seán Fitzgerald, Shunli Wang, Daying Dai, Dennis H. Murphree, Abhay Pandit, Andrew Douglas, Asim Rizvi, Ramanathan Kadirvel, Michael Gilvarry, Ray McCarthy, Manuel Stritt, Matthew J. Gounis, Waleed Brinjikji, David F. Kallmes, Karen M. Doyle

**Affiliations:** 1 CÚRAM–Centre for Research in Medical Devices, National University of Ireland Galway, Galway, Ireland; 2 Department of Radiology, Mayo Clinic, Rochester, Minnesota, United States of America; 3 Department of Physiology, National University of Ireland Galway, Galway, Ireland; 4 Department of Pathology, Shanghai East Hospital, Tongji University, Shanghai, China; 5 Department of Health Sciences Research, Mayo Clinic, Rochester, Minnesota, United States of America; 6 Cerenovus, Ballybrit, Galway, Ireland; 7 Orbit Image Analysis, Binningen, Switzerland; 8 Department of Radiology, New England Center for Stroke Research, University of Massachusetts Medical School, Worcester, Massachusetts, United States of America; Bielefeld University, GERMANY

## Abstract

Our aim was to assess the utility of a novel machine learning software (Orbit Image Analysis) in the histological quantification of acute ischemic stroke (AIS) clots. We analyzed 50 AIS blood clots retrieved using mechanical thrombectomy procedures. Following H&E staining, quantification of clot components was performed by two different methods: a pathologist using a reference standard method (Adobe Photoshop CC) and an experienced researcher using Orbit Image Analysis. Following quantification, the clots were categorized into 3 types: RBC dominant (≥60% RBCs), Mixed and Fibrin dominant (≥60% Fibrin). Correlations between clot composition and Hounsfield Units density on Computed Tomography (CT) were assessed. There was a significant correlation between the components of clots as quantified by the Orbit Image Analysis algorithm and the reference standard approach (*ρ* = 0.944**, *p* < 0.001, *n* = 150). A significant relationship was found between clot composition (RBC-Rich, Mixed, Fibrin-Rich) and the presence of a Hyperdense artery sign using the algorithmic method (*X*^*2*^(2) = 6.712, *p* = 0.035*) but not using the reference standard method (*X*^*2*^(2) = 3.924, *p* = 0.141). Orbit Image Analysis machine learning software can be used for the histological quantification of AIS clots, reproducibly generating composition analyses similar to current reference standard methods.

## Introduction

The use of endovascular therapy (ET) to treat Acute Ischemic Stroke (AIS) is now common practice following multiple positive large clinical trials [1–5]. Aside from the demonstrated clinical benefits of increased recanalization rates and improved functional outcomes, the use of ET also affords researchers the opportunity to study the histopathological composition of AIS clots, which was not previously possible when patients were treated with thrombolytic drugs alone. The histopathological composition of AIS clots can provide crucial information regarding clot pathogenesis which may inform future clinical management of patient care. For example, histopathological analysis of clots can identify patients who had platelet-rich clots, and who might potentially benefit from antiplatelet therapy in order to prevent recurrent strokes [6]. Conversely, the identification of patients who did not have platelet-rich clots could be spared the potential increased risk of bleeding often associated with antiplatelet therapy [7].

Whilst pathological assessment of histology remains the gold standard for diagnosis and prognosis of disease, the advent of digital pathology, whole slide scanning and computational image analysis may help to address difficulties related to the manual assessment of histology. Inter-observer variability, low-reproducibility and inaccurate quantitative assessment are examples of errors that may be addressed by machine learning using digital pathology [8]. Presently, most digital pathological quantification is done using software that segments the image based on color thresholding, whereby the user defines specific pixel density cut-off levels per cell/tissue type. Adobe Photoshop and Image J are commonly used programs for histopathological quantification [9–12]. In contrast, the software program Orbit Image Analysis addresses tissue quantification via a machine learning approach, wherein numeric features are computed and then used as input for a prediction algorithm known as a support vector machine (SVM) [13]. These features and their accompanying predictions allow the software to incorporate the structure of the tissue into its quantification assessment rather than relying on the pixel intensity alone. Our aim was to assess the utility of Orbit Image Analysis in the histological quantification of AIS blood clots in comparison to a currently used reference standard.

## Materials and methods

### Patient selection and clinical data

This study is a dual-institutional, HIPAA compliant and institutional review board approved (Mayo Clinic Rochester and University of Massachusetts Memorial Medical Center), investigational study of AIS patients who have undergone clot retrieval using mechanical thrombectomy techniques. A waiver of consent was granted for all patients included in the study. Inclusion criteria include 1) adult patients (>18 years), 2) having undergone mechanical thrombectomy with retrieval of clot material who 3) have clot available for histopathological analysis. AIS patients who did not have clot available for analysis were excluded from the study. Clinical data for included patients was reported on a single-page data abstraction form. The data abstraction form contains information regarding demographics, use of intravenous or intra-arterial recombinant tissue plasminogen activator (rtPA), occlusion location and suspected stroke etiology, imaging appearance of the clot on Computed Tomography (CT), devices and technique used, number of passes needed to achieve final revascularization status and final angiographic outcome (Table 1). 10 patients were excluded from the study due to incomplete data. Fifty patients were included in the study, 25 treated at Mayo Clinic and 25 at treated University of Massachusetts Memorial Medical Centre.

**Table 1:** Clinical details of patient cohort.

		Number of Patients *(n = 50)*	(%)
**Age:**			
Median	67		
Range	20–91		
**Sex:**			
Male		22	44%
Female		28	56%
**Site:**			
ICA		14	28%
MCA: M1 & M2		38	76%
ACA		1	2%
Basilar		2	4%
Vertebral		1	2%
PCA		1	2%
SCA		1	2%
**rtPA:**			
Yes		23	46%
No		27	54%
**No of Passes:**			
Mean	2.36		
1		20	40%
2		10	20%
3		11	22%
4		4	8%
5+		5	10%
**Final TICI Score:**			
2a		1	2%
2b		30	60%
3		19	38%
**Mean HU:**			
All Clots	54.0	50	100%
Cardioembolic	53.6	30	60%
Large Artery	49.4	11	22%
Unknown	53.7	4	8%
Other	69.5	5	10%
**Stentriever Used:**			
Yes		34	68%
No		16	32%
**Aspiration Only:**			
Yes		16	32%
No		34	68%

ICA: Internal Carotid Artery; MCA: Middle Cerebral Artery; ACA: Anterior Communicating Artery; PCA: Posterior Cerebral Artery; SCA: Superior Cerebellar Artery; rtPA: recombinant tissue plasminogen activator; TICI Score: Thrombolysis in Cerebral Infarction (TICI) Score; HU: Hounsfield Units.

### Computed tomography imaging

Prior to endovascular treatment, each patient had a non-contrast CT scan performed. Using a research imaging archive system, expert readers at each site evaluated the mean and maximum clot attenuation as measured by the placement of regions of interest (ROIs) along the clot. A positive Hyperdense Artery Sign (HAS) was defined as ≥50 Hounsfield Units (HU).

### Clot collection, processing and histology

On retrieval, the clot was immediately fixed in 10% phosphate-buffered formalin for up to 24 hours. Clots were then placed in formalin for 1 hour, followed by 70% alcohol for 1 hour, 2 changes of 95% alcohol (1 hour each), and 2 changes of 100% alcohol (1 hour each). Next, specimens were placed in 2 changes of Xylene (1 hour and 45 mins each), followed by 2 changes of liquid paraffin (1 hour and 45 mins each). Following tissue processing the clots were then embedded in paraffin blocks. At the University Of Massachusetts Memorial Medical Center the clots were then shipped to the Mayo Clinic histology core lab for analysis. In order to minimize the superposition of different cells and tissues, FFPE clot material was cut into thin slices (3–5μm). Two serial sections were placed on each slide. Three slides from each clot were stained with Hematoxylin and Eosin (H&E). Briefly, slides were deparaffinised and hydrated in water, followed by staining with hematoxylin (Sigma Aldrich) for 5 minutes. Slides were stained with Eosin-Y (Thermo Scientific) for 1 minute and then quickly dehydrated in graded alcohol solutions, cleared with xylene, and mounted with EZ-Mount (ThermoShandon). In order to minimize intraday staining variability, slides were stained in large batches, with freshly prepared reagents.

A trained pathologist assessed the H&E staining quality, confirming that the composition of the clots could be characterized into 3 major components (RBCs, WBCs and Fibrin) based on the morphologic element staining with red, blue and pink color respectively. A representative slide for each clot that was then sent for whole slide scanning at 20x magnification using standard scan settings (Aperio ScansScope AT Turbo, Leica Biosystems). Histologic characterization was performed on the digital slide by an expert pathologist and an experienced researcher in accordance with the guidelines put forth by the Clot Summit Group [14]. The pathologist and researcher analyzed the slides independently. Following quantification, the clots were grouped into 3 sub-types based on the ratio of each clot component 1; RBC-rich clots (≥60% RBCs), 2; mixed clots (RBCs proportion equal to fibrin), and 3; fibrin-rich clots (≥60% fibrin) in accordance with previous studies [15, 16].

### Adobe Photoshop software

**Fig 1:**
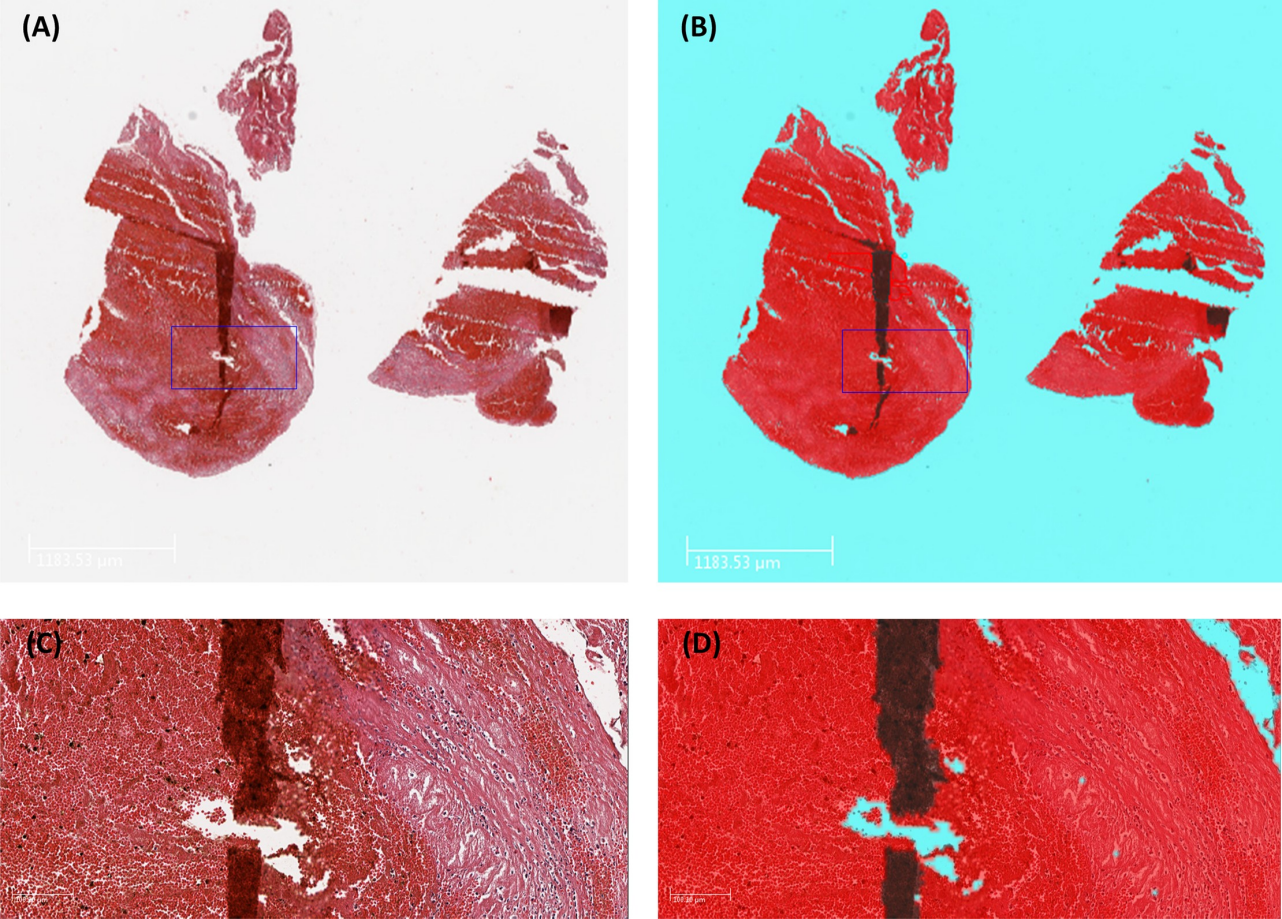
Background and artefact detection. (A&C) Histopathological staining of an AIS clot with H&E stain, (1X & 10X respectively). A fold is clearly visible on the tissue. (B&D) Output image from Orbit Image Analysis software following the use of an Exclusion model. Background, artefact and tissue are depicted in blue, black and red respectively. Orbit Image Analysis correctly identifies and excludes the folds on the tissue (black).

The same pathologist performed the component segmentation on each image through color (pixel) thresholding of each component using a reference standard technique (Adobe Photoshop CC Software, Version: 2017 Adobe systems, San Jose, CA, USA). AIS clots by their nature are often difficult to cut using a microtome, occasionally leading to minor folds (artefact) on the tissue as can be seen in Fig 1A & 1C below. This folding can lead to inaccuracies in the quantification of clot composition. Therefore prior to segmentation, these areas of artefact are first manually cropped-out of the digital slide by the pathologist. Next, a region of a particular color representing a certain cell or tissue type is selected by the pathologist on the digital slide. As only a single representative region can be chosen for each cell/tissue type, it is important to select a region that is an accurate representative of that cell or tissue type. The software then determines the range or threshold of pixels within the marked region and calculates the number of pixels within this threshold range that are present in the entire image, as well as the total number of pixels within the image. The pathologist takes note of these pixel values and manually calculates the percentage of the selected cell/tissue type by expressing it as a percentage of the total number of pixels within the image. This process is repeated for each component within the clot (e.g. RBCs, WBCs and Fibrin). This method is then repeated for every slide that is included in the study.

### Orbit Image Analysis

Quantification of clot components was also independently performed on the set of slides described above by a researcher, with over 5 years of experience in histopathological analysis, using Orbit Image Analysis (www.orbit.bio) as per the Standard Operating Procedure (S1 and S2 Appendices). Orbit Image Analysis is a free open source software package that incorporates machine learning to quantify tissue content [13]. This software package includes image analysis algorithms designed for image segmentation, classification and quantification that are described in detail previously [13, 17, 18]. Briefly, these models are based on a sliding window feature extraction approach; for a region surrounding each pixel, a set of six hand-crafted numeric features is calculated. These features are simple scalars such as the maximum, minimum, variance or edge factor pixel values within the window and this set of six features is then associated with the label annotated by the user. These features describe the structure of the underlying tissue or other biological sample and are used as an input for a Support Vector Machine (SVM). The support vector machine is trained using the numeric features as predictors to classify the center pixel label to discriminate regions within the image [19]. Once trained these models are applied to the entire slide, classifying each pixel into a specific feature type as depicted in Fig 1. To account for intra-slide staining variability, a training set of representative H&E stained images is used to train the machine learning model.

The training process begins with the user training an Exclusion model on Orbit Image Analysis. The Exclusion model is trained to identify the areas of the whole slide scan that are to be included for analysis (e.g. clot tissue) and those that are to be excluded (e.g. background and artefact). The user defines the components that they wish to include and exclude (e.g. Background (Exclude), Tissue (Include) and Artifact (Exclude) and annotates multiple regions of each component on the digital slide scans of the training set of images (≥5 images). The model is then trained to recognize Background, Tissue and Artefact as distinct from each other. The resulting segmentation map is then displayed to the user who evaluates it for accuracy, as can be seen in Fig 1. This Exclusion model is then be applied to the validation set of slides to ensure that it is accurate (≥5 images). Once the accuracy is validated by the user, this model is then saved as the Exclusion model.

Next the user trains the Classification model. The user begins by defining the cell/tissue types that they wish to characterize, which in this case is RBCs, WBCs and Fibrin. The user annotates multiple regions of each specific cell/tissue type on the on digital slide scans of the training set of images. These regions can be as specific as individual cells for RBCs and WBCs or include larger areas of tissue, such as Fibrin strands. The user can use several different shaped tools to annotate the slides including circles, rectangles and polygons. The classification model is then trained to recognize each of the annotated cell/tissue types on the training set of images (≥5 images). The user validates the accuracy of the Classification model on the validation set of images (≥5 images). This training and evaluation procedure can be repeated, adding more annotated cell/tissue until an accurate tissue classification model is developed. At least one slide from each staining batch to be tested should be included in each of the Test and Validation sets of images. Once validated, the model is saved as the Classification model.

**Fig 2:**
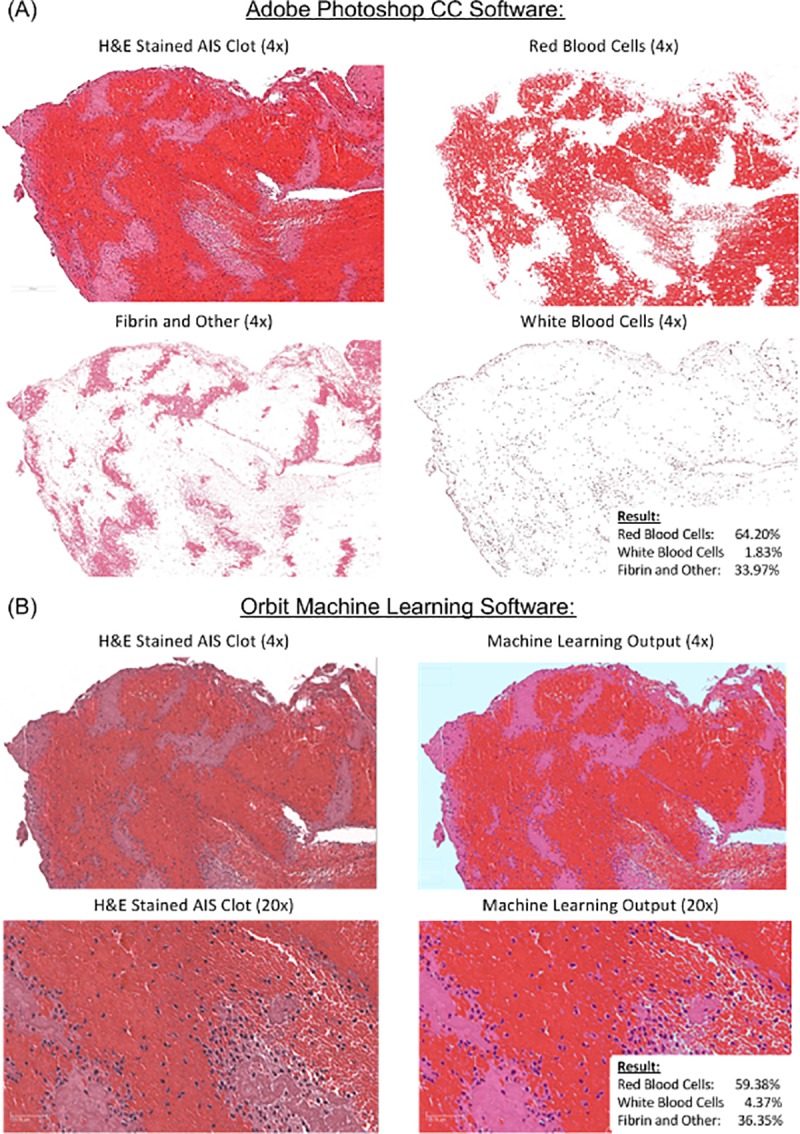
Adobe Photoshop versus Orbit Image Analysis. (A) Histopathological analysis of the H&E stained AIS clot was performed and clot composition was quantified via standard thresholding techniques using Adobe Photoshop CC. (B) Following representative cell labelling by an expert pathologist, Orbit Image Analysis was trained to recognize various cell and tissue types and clot composition was quantified. There was a significant correlation between clots quantified using Adobe Photoshop and Orbit Image Analysis (ρ = 0.944, p < 0.001**).

Once trained separately, the Exclusion and Classification models are then combined to form the overall ‘Main’ model used for histological quantification. Only areas marked for inclusion (i.e. clot tissue) on the Exclusion model will be classified using the Classification model. The algorithm is then tested on a validation set of images and the output from the validation set of images is visually inspected for accuracy (Fig 1B & 1D and Fig 2B). Once the user is confident that the algorithm is accurate, it is applied to the test set of images in a batch mode which runs autonomously. The model then works automatically to segment, classify and quantify each component within each slide being analyzed. The result is a quantitative assessment of the proportion of RBCs, WBCs and Fibrin within each clot (Fig 2B).

### Statistical analysis

All statistical correlations were assessed using IBM SPSS Statistics 22 and Graphpad Prism 8. Bland-Altman plots were used to analyze the agreement between the two analysis methods; the differences between the two techniques (Orbit and Adobe) was plotted against the averages of the two techniques. Rank correlations between Orbit Image Analysis and the reference standard were also assessed using a Spearman Rho correlation. Correlations between clot composition and Hounsfield Units (HU) density on CT were assessed using the Chi-Squared test.

## Results

### Patient cohort

In total 50 patients with a diagnosis of AIS and treated with mechanical thrombectomy met the inclusion criteria and were included in the study. Table 1 shows the clinical demographics of the patient cohort. The median age of the patients was 68 years (range 20–91 years). The cohort included 22 male and 28 female. Forty-six percent of patients had been treated with t-PA prior to mechanical intervention. The majority of cases had an ICA or MCA occlusion (26% & 76% respectively). Several of the patients had occlusions in multiple sites (14%, data not shown). The average number of passes per patient was 2.38 and the mean Hounsfield Unit density of the clots was 54.0. Stentriever devices were used in 68% of patients, whilst aspiration alone was used to treat the remaining 32% of patients. TICI 2b/3 was achieved in 98% of patients treated across both sites.

### Histopathological characterization: Orbit Image Analysis vs reference standard analysis

**Fig 3:**
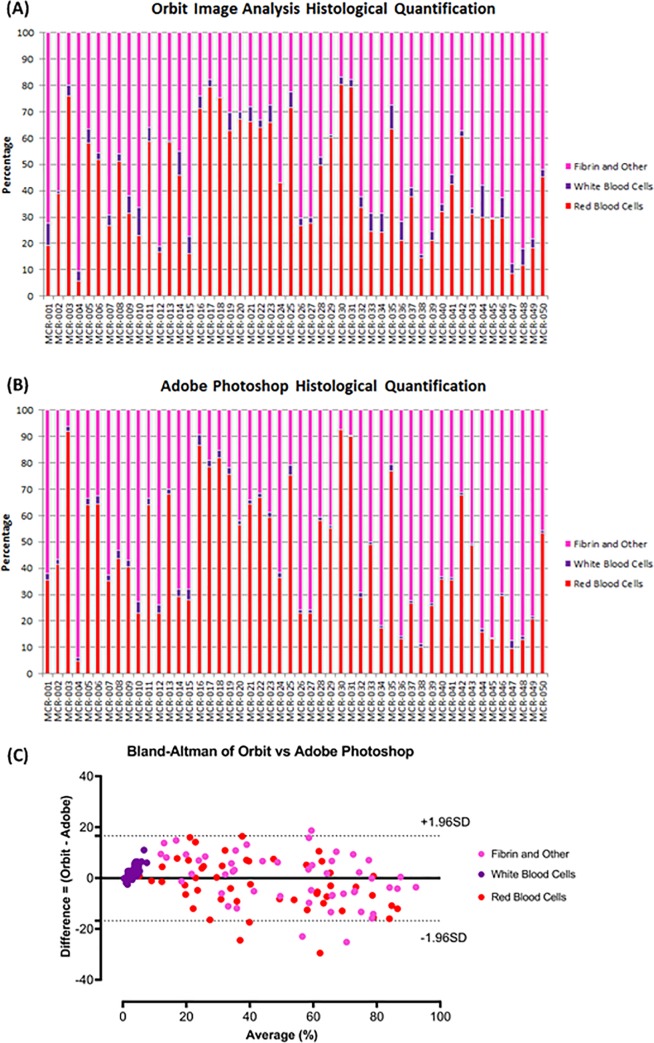
Quantified histological clot composition. Graphical representation of the histological composition of 50 AIS clots following quantification using Orbit Image Analysis software (A) and Adobe Photoshop Software (B). The results from both Orbit Image Analysis and Adobe Photoshop are similar for each patient in the study. (C) The Bland-Altman plot demonstrated that there was good agreement between Orbit Image Analysis and Adobe Photoshop, the bias was -0.01±8.51 (Mean±SD) and the limits of agreement were -16.8 to 16.6. Red = Red Blood Cells, Purple = White Blood Cells and Pink = Fibrin as per the H&E stain.

The pathologist quantified the percentage of RBCs, WBCs and Fibrin in each clot using the reference standard approach (Adobe Photoshop). A representative example of each is presented in Fig 2 and the quantification results using both Orbit Image Analysis and Adobe Photoshop are depicted in Fig 3. The researcher also quantified the percentage of RBCs, WBCs and Fibrin in each clot using Orbit Image Analysis. A representative example of each is presented in Fig 2 and the quantification results using both Orbit Image Analysis and Adobe Photoshop are depicted in Fig 3. The Bland-Altman plot showed the mean bias ±SD between Orbit Image Analysis and the reference standard approach (Adobe Photoshop Image-Pro) was -0.01±8.51 (Mean±SD) and the limits of agreement were -16.8 to 16.6 (Fig 3). There was a significant correlation between Orbit Image Analysis and the reference standard approach (Adobe Photoshop Image-Pro) when used to quantify H&E stained AIS clots (*ρ* = 0.944, *p* < 0.001**). Both Orbit Image Analysis and the reference standard approach (Adobe Photoshop) are capable of detecting and quantifying each component (RBCs, WBCs and Fibrin) within a clot and there was good agreement between the methods used.

### Correlation between clot composition and Hounsfield Units

**Fig 4:**
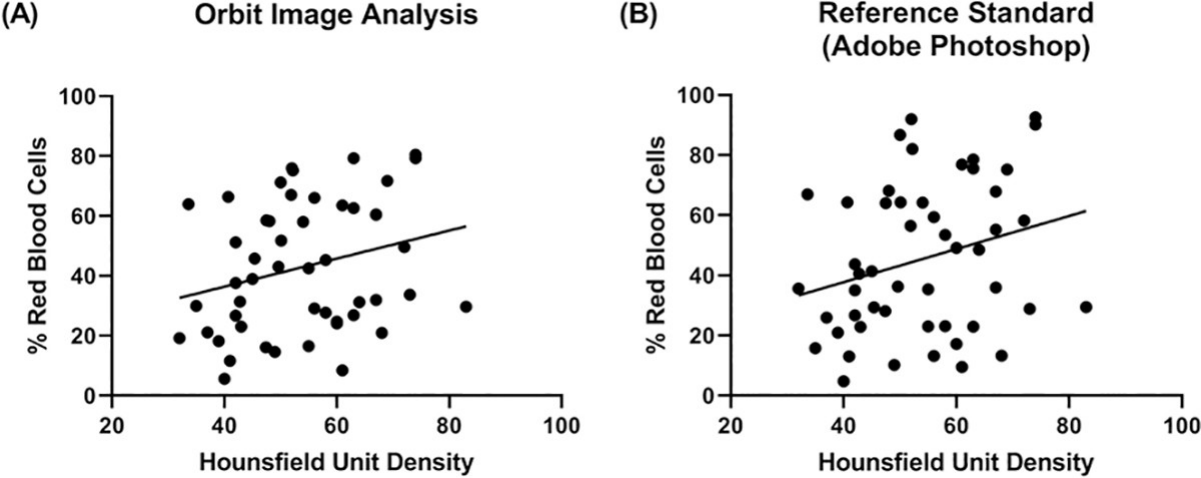
Red blood cell composition versus Hounsfield Units. Corresponding % Red Blood Cell composition versus Mean Hounsfield Unit when measured by (A) Orbit Image Analysis and (B) Adobe Photoshop. Mean Hounsfield Unit values are taken from the non-contrast CT scans performed prior to endovascular treatment for each of the 50 clots. As the trend lines suggest, there is a significant positive correlation between the percentage of Red Blood Cells and Mean Hounsfield Unit Densities on non-contrast CT scans when measured using the Orbit Image Analysis software (ρ = 0.291*, p = 0.040*) and Adobe Photoshop (ρ = 0.280*, p = 0.049*).

**Fig 5:**
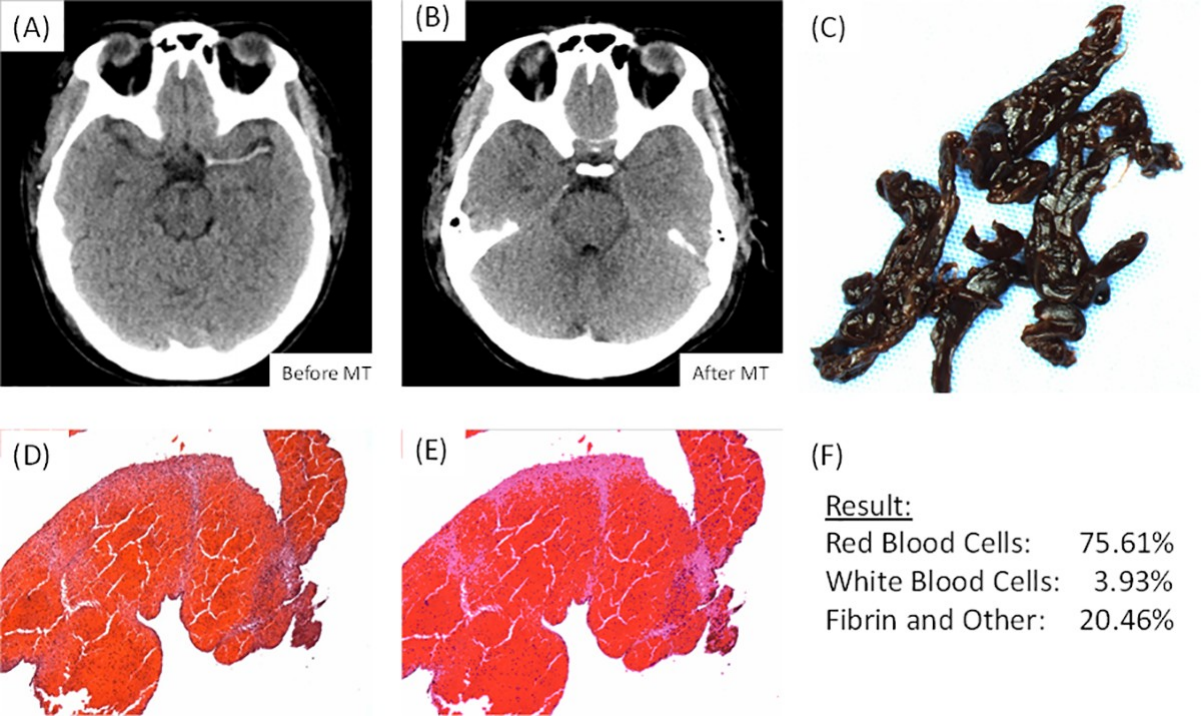
Patient example. This is an example of a patient who presented at Mayo Clinic, Rochester, with a clot in the M1 segment of their Left Middle Cerebral Artery. (A) Is an image taken from the non-contrast CT scan of the patient prior to the procedure demonstrating the presence of the clot (B) Is an image taken from the non-contrast CT scan of the patient after the procedure demonstrating successful removal of the clot (C) Is a gross photo of the clot that was removed (1.25x). (D) Is a high-powered image (100x) of the corresponding (H&E) stained slide. (E) Is an image of the output from the Orbit software following quantification of the H&E stained slide. (F) Is the results of the histological quantification.

There was a positive correlation between the percentage of Red Blood Cells and Mean Hounsfield Unit densities on non-contrast CT scans when measured using the Orbit Image Analysis software (ρ = 0.291, p = 0.040*) and Adobe Photoshop (ρ = 0.280, p = 0.049*; Fig 4). A patient example is illustrated in Fig 5. A significant relationship was found between clot composition (RBC-Rich, Mixed, Fibrin-Rich) and the presence of a HAS sign, (*X*^*2*^(2) = 6.712, *p* = 0.035*) when quantified using the Orbit software. There was also a positive correlation between Red Blood cell rich clots (≥60 RBCs) and the presence of a Hyperdense artery sign on CT (X2(2) = 6.349, p = 0.012*) using Orbit image analysis. Contrastingly, no significant relationship was found between clot composition (RBC-Rich, Mixed, Fibrin-Rich) and the presence of a HAS sign, (X2(2) = 3.924, p = 0.141) or between Red Blood cell rich clots (≥60 RBCs) and the presence of a Hyperdense artery sign on CT (*X*^*2*^(2) = 2.206, *p* = 0.138) when using the Adobe Photoshop software, although a similar trend is evident.

## Discussion

In this study we demonstrated that the output of Orbit Image Analysis’ internal machine learning algorithm correlates with the conventional histological reference standard quantification method in its ability to detect and quantify the composition of AIS blood clots. Once trained, these SVM-based models can be applied to multiple slides in a fully automated manner (Batch Mode) making the process of histological characterization and quantification faster and less labor-intensive [17]. Importantly, the trained models can be saved and reapplied at a later date resulting in no inter-day variability. These findings are important because use of this software will help to address common errors in histological quantification such as inter-observer variability, low-reproducibility and inaccurate manual quantitative assessment [8]. The method described herein refers to the quantification of AIS Clots stained with Hematoxylin and Eosin, but the process can equally be employed for the characterization and quantification of other histologically and immunohistochemically stained slides and in other tissue types.

Manual assessment of histology remains the gold standard for primary diagnosis of many serious diseases such as cancer, yet it is subject to low accuracy and reproducibility particularly in the quantitative estimation of composition [20]. The advent of high-resolution whole slide scanners and the process of digitization of tissue slides have resulted in a significant advancement in the field of digital pathology and consequential improvement in digital pathology image analysis [8, 21]. To date, many image analysis software packages including Aperio, Lucia, Metaview, Metamorph, ImageJ, Scion, Adobe Photoshop and Image Pro Plus have been used for the analysis of digital pathology images. Each image analysis tool has its own advantages and limitations, yet no image analysis software package has been accepted as the gold-standard approach. Upon reviewing the aforementioned image analysis tools, *Prasad et al* (2012)., concluded that there is good scope for development of freely available software for staining intensity quantification, which a medical researcher could easily use without requiring high level computer skills [10].

Orbit Image Analysis is a free, user friendly, open source software with ability to quantify big images like whole slide scans and has many built-in image analysis algorithms such as tissue quantification using machine learning techniques, object/cell segmentation, and object classification that are compatible with whole slide scan images which can be used by researchers with minimal training. Firstly, an image quality analysis should be performed prior to analysis [22]. By identifying and eliminating artefact on the slide, the exclusion model increases the accuracy of the quantification by removing that which may otherwise be wrongly classified. The use of an exclusion model also reduces overall processing time as the background and artefact are excluded from further processing. Importantly, Orbit Image Analysis allows for the annotation of multiple regions per cell/tissue type and the regions can be as specific as an individual cell. This is not possible with the reference standard method used, where only one region can be selected. Notably, this study demonstrated statistically significant correlations involving clot composition when quantified using Orbit that were not identified by the current reference standard approach which may be due to a more accurate clot characterization being achieved using Orbit Image Analysis than the current reference standard.

The Hematoxylin and Eosin stain was chosen for this study as it has been described as the cornerstone of anatomical pathology diagnosis and is the most widely used histological stain [23, 24]. The Bland-Altman analysis demonstrated that there was good agreement between Orbit Image Analysis and the reference standard method (Adobe Photoshop) when quantifying the major components of AIS clots following H&E staining. The limits of agreement were relatively small at -16.8 to 16.6 and the bias was -0.01±8.51 (Mean±SD). The reference standard is not currently used as a clinical tool and is not without flaw, it is a subjective method that may be influenced by inter-user variability and issues related to reproducibility. Therefore, any minor discordance between Orbit Image Analysis and Adobe Photoshop may be due in part to the limitations of the reference standard approach itself.

Previous studies have suggested that the Hounsfield unit density of an AIS clot on a non-contrast CT can identify the the composition of the occlusive clot, with Red Blood cells rich clots being Hyperdense and platelet-rich clots being isodense [15, 25–28]. In accordance with these previous findings, our study found a relationship between clot composition (RBC-Rich, Mixed, Fibrin-Rich) and the presence of a HAS sign when quantified using the Orbit image analysis software. A significant positive correlation between Red Blood cell rich clots (≥60% RBCs) and the presence of a Hyperdense artery sign on CT was observed. Measuring the Hounsfield unit density of the clot on the non-contrast CT prior to endovascular intervention may help to inform the clinician of the clot type (RBC-Rich Vs Other) and thus may help to determine the most appropriate treatment strategy for that clot as clot composition is known to influence the outcome of various endovascular therapies [27, 29–31]. However, further studies on larger data sets and standardization of diagnostic imaging and HU measurement are required before these conclusions can be confirmed and impact the treatment of patients.

The field of digital pathology is in its infancy and therefore guidelines for digital pathology need to be followed closely to ensure compliance and reduce variability between studies [32]. One of the main challenges in the field of digital pathology image analysis is color and intensity variations induced by slight disparities in slide preparation and staining. In an effort to minimize batch to batch variability, we conducted staining in large batches, using freshly prepared reagents and stuck rigidly to the methodology in the standard operating procedure. The use of an Autostainer can help to further reduce potential minor variations. The robustness of the trained algorithms across different batches has yet to be assessed quantitatively, but early indications suggest that it is fairly robust. Batch to batch variability cannot be assessed using the reference standard technique as each image has to be analyzed individually.

## Supporting information

S1 Appendix Orbit Image Analysis standard operating procedure for histological quantification. (PDF)

S2 Appendix Manuscript data. (XLSX)
