# Supplementary material for: Orbit image analysis machine learning software can be used for the histological quantification of acute ischemic stroke blood clots
Source: PLoS One. 2019 Dec 5;14(12):e0225841. doi: 10.1371/journal.pone.0225841 (PMC6894878; doi:10.1371/journal.pone.0225841)
Supplement: S1 Appendix — (PDF) [file pone.0225841.s001.pdf]

Title: Machine learning techniques accurately quantify the histological composition of Acute Ischemic Stroke blood clots

Authors: Seán Fitzgerald*[,1,2,3], Shunli Wang[2,4], Daying Dai[2], Dennis H. Murphree Jr.[5], Abhay Pandit[1], Andrew Douglas[1,3], Asim Rizvi[2], Ramanathan Kadirvel[2], Michael Gilvarry[6], Ray McCarthy[6], Manuel Stritt[7], Matthew J. Gounis[8], Waleed Brinjikji[2], David F. Kallmes[2], Karen M. Doyle[1,3].

[1]CÚRAM–Centre for Research in Medical Devices, National University of Ireland Galway, Galway, Ireland, [2]Department of Radiology, Mayo Clinic, Rochester, MN 55905, USA, [3]Physiology Department, National University of Ireland, Galway, University Road, Galway, Ireland, [4]Department of Pathology, Shanghai East Hospital, Tongji University, Shanghai, China, [5]Department of Health Sciences Research, Mayo Clinic, Rochester, MN 55905, USA, [6]Cerenovus, Galway, Ireland, [7]Kernmattstrasse 22, CH-4102 Binningen, Switzerland, [8]Department of Radiology, New England Center for Stroke Research, University of Massachusetts Medical School, 01655, Worcester, Massachusetts, USA.

Supplementary File 1:

Orbit Image Analysis Standard Operating Procedure for Histological Quantification.

Orbit Image Analysis Standard Operating Procedure for Histology Quantification:

Orbit is free to download from https://www.orbit.bio/ and there are versions available for Windows, Linux and Mac.

For the purposes of this SOP I am going to use H&E as the stain and Red Blood Cells, White Blood Cells and Fibrin as the tissue types that I wish to quantify. However this can be adapted to different stains and different cell/tissue types as necessary.

1). Click on the desktop icon to Launch Orbit.

2). Sometimes a popup comes up and just click continue.

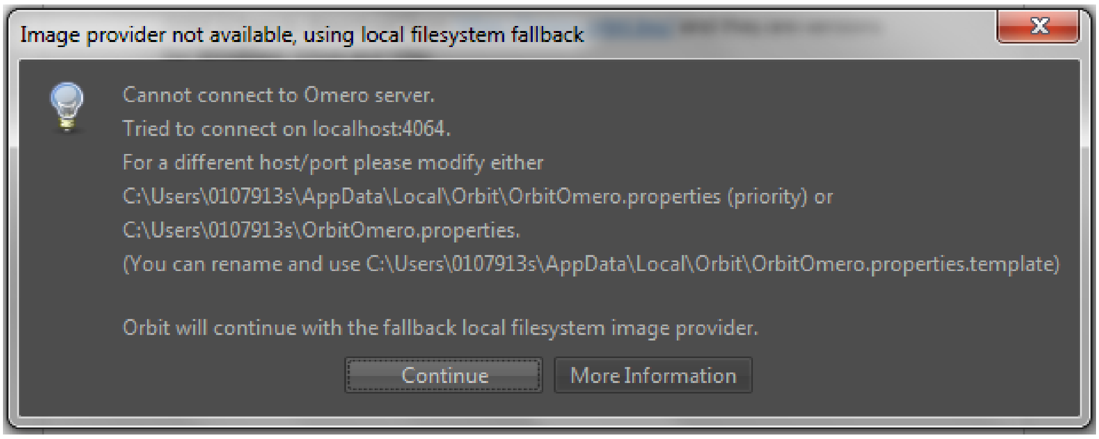

3). Click on the Image Tab and then click on Open Image from File System.

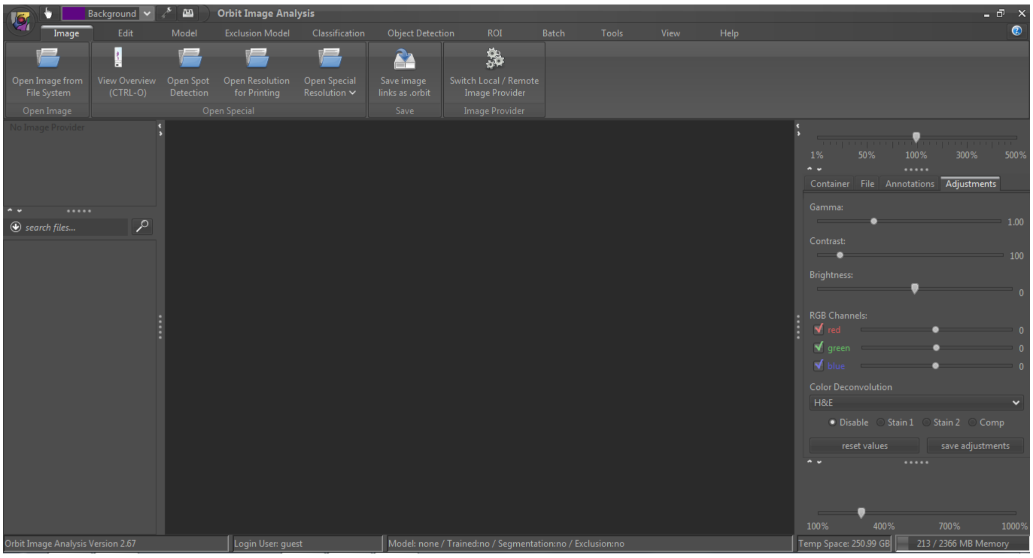

4). Browse to the folder that the file/image to be analysed is in. If you are working off a static Image (.jpeg, .tiff, .png, etc.) you can just select it and it will open.

*However if you are working off a whole slide scan file (.svn, .vsi, etc.) you need to use the Series tab to find the correct series for analysis. E.g. Series 1 is usually just the Label.

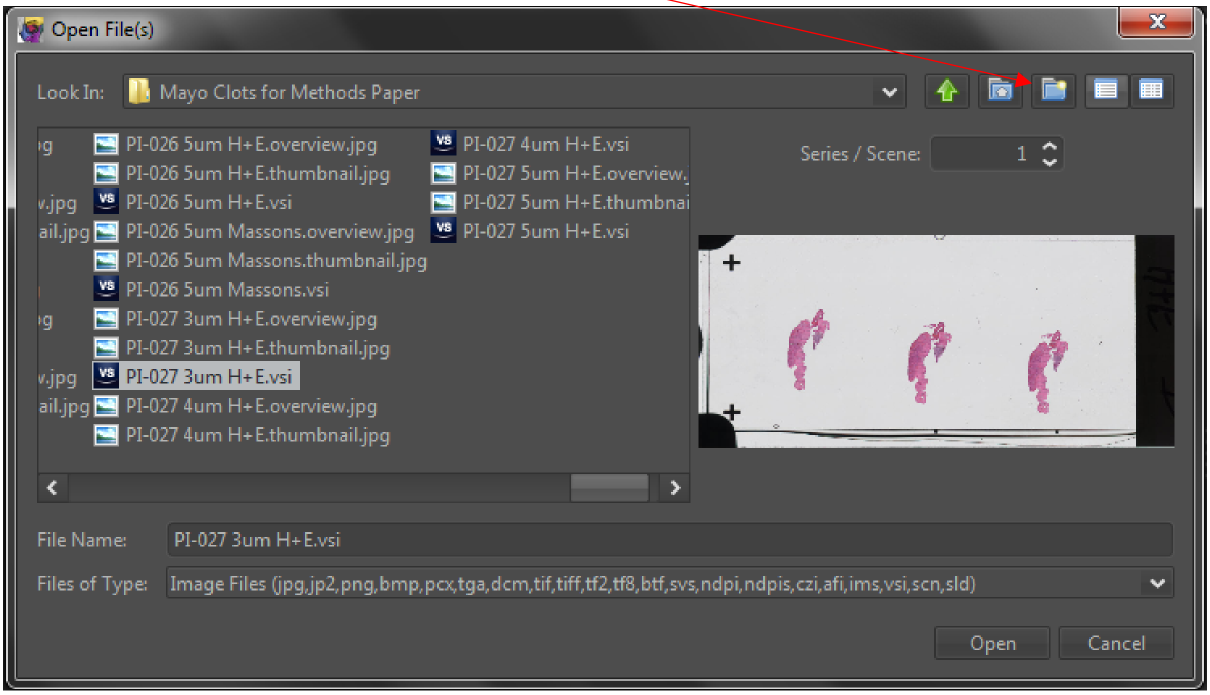

Click Open to open the image. Zoom in to make sure the definition is good.

5). The brightness and contrast of the image can be adjusted on the right if necessary.

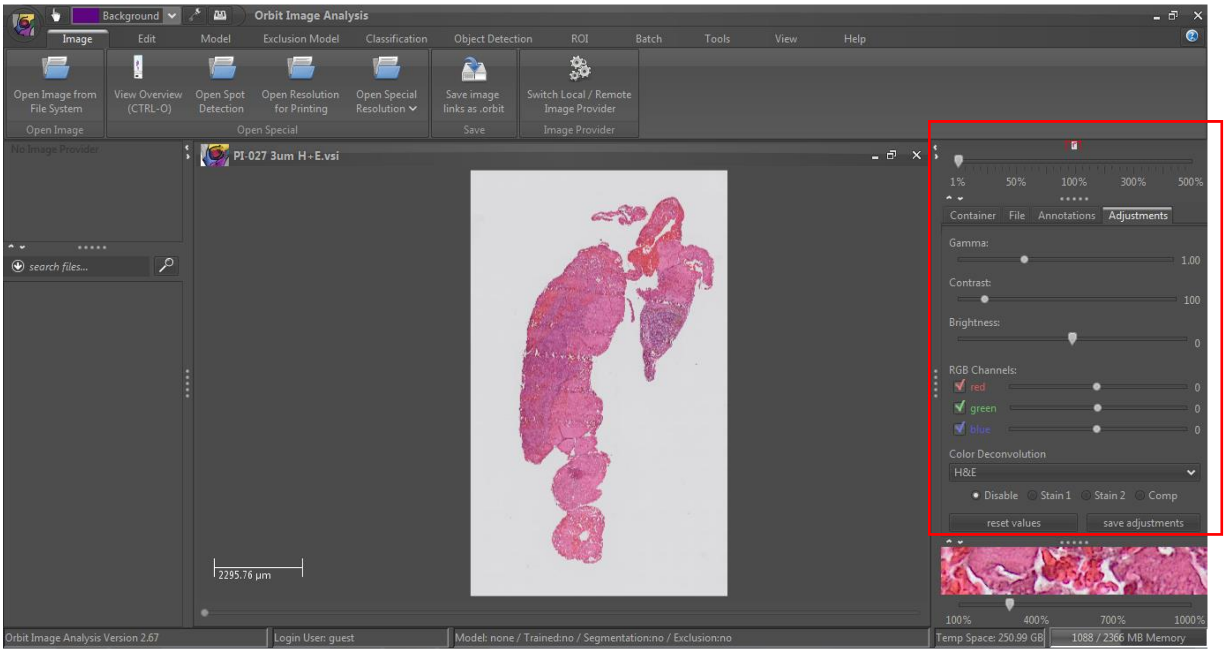

*6). **Exclusion Model:** This step allows you to define the tissue from background and also to mark areas of artefact which will be excluded from the quantification, increasing the accuracy of the model and quantification results.

Click on the Exclusion Model Tab, and then click on the spanner icon to setup classes 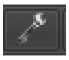

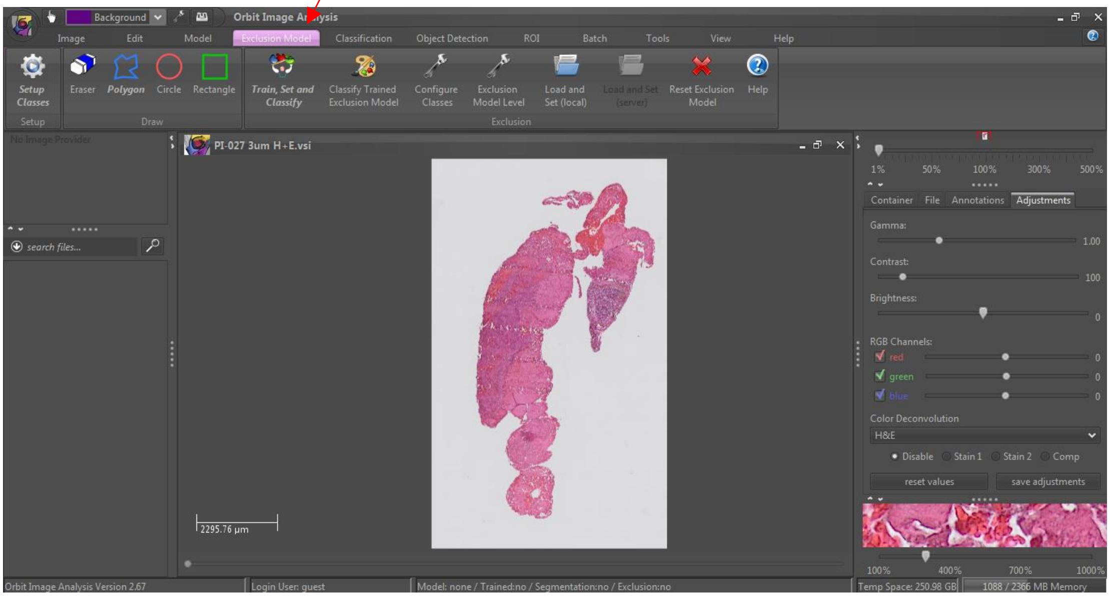

7). Rename the Classes as you wish. (E.g. Background, Tissue and Artefact). You can also adjust the colours that you use to represent each class.

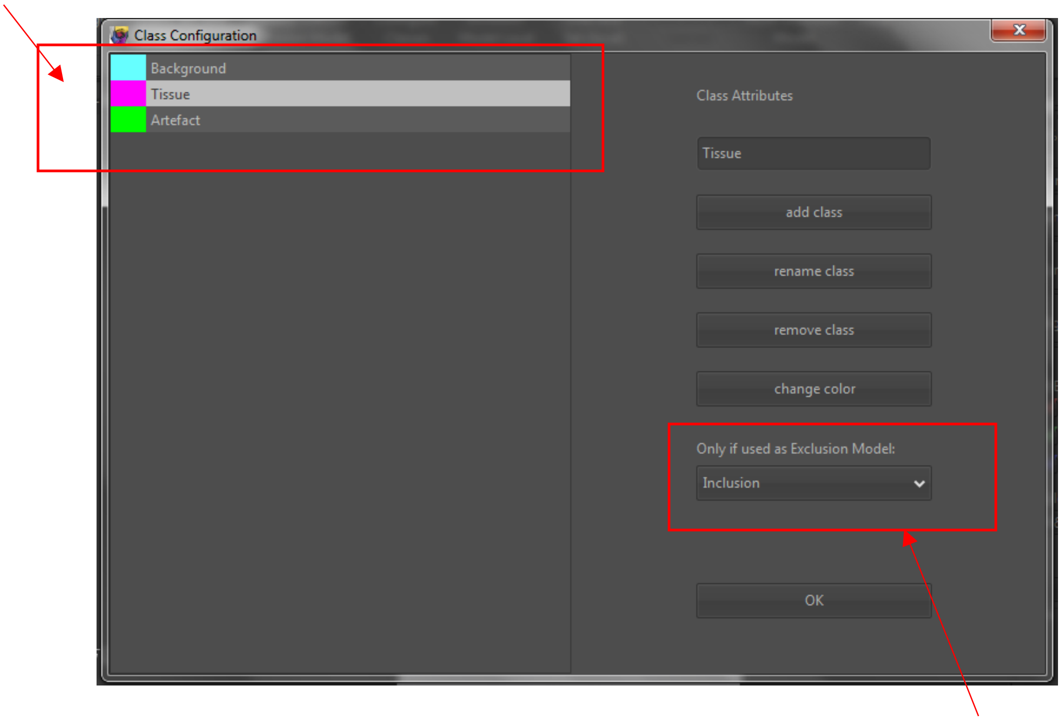

The important thing is to define each class as Inclusion or Exclusion. E.g. Background and Artefact are Exclusion because you don't want to quantify them. Tissue is Inclusion and you want to quantify the tissue. Click ok.

8). Whist on the Exclusion Tab, Select Background. Use the different tools (Circle, rectangle, Polygon) to mark areas, of background on your image. (You can zoom in and navigate around your image by clicking on the hand tool 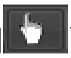). For example click on the Circle, move to an area of background and mark an area of background by left-clicking on the mouse.

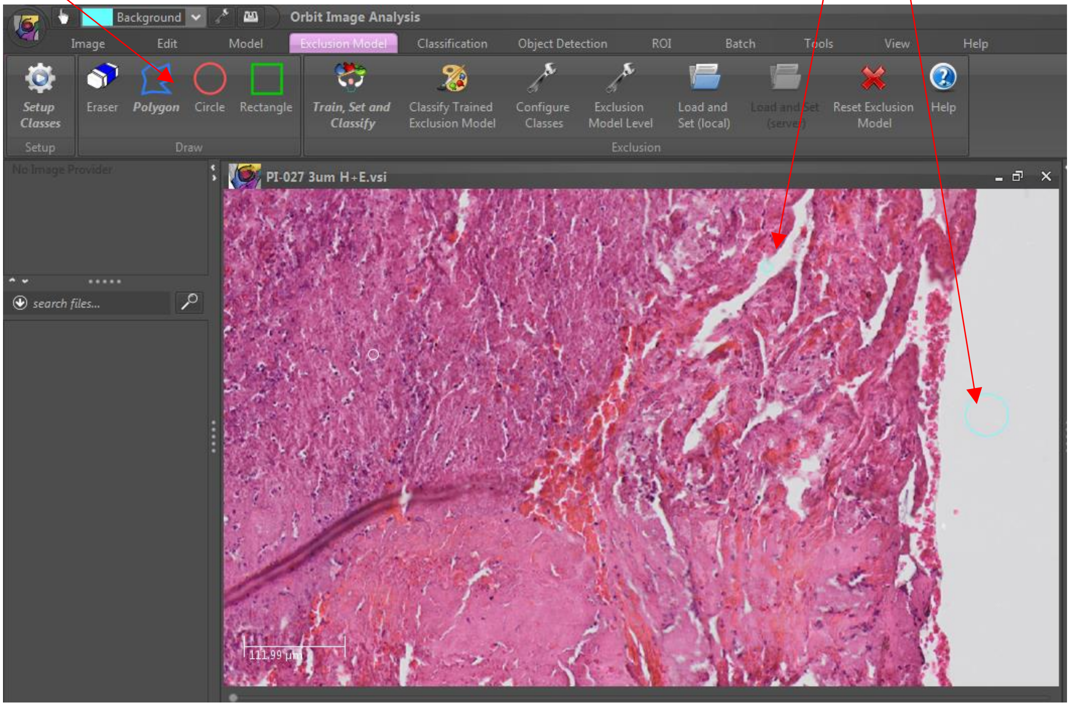

9). Now Change to the Tissue tab and repeat the process to mark areas of tissues for inclusion. It is a good idea to mark different areas within the tissue.

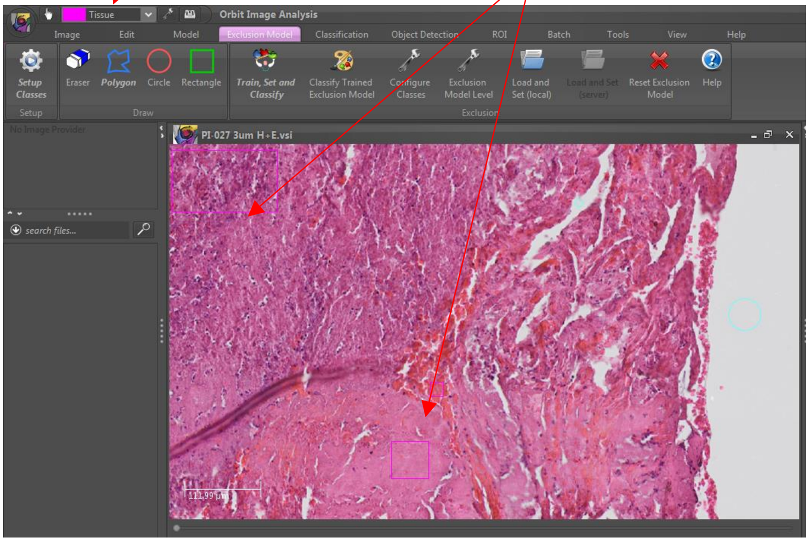

10). Now Change to the Artefact and repeat the process to mark areas of artefact for Exclusion. *Make sure not to touch areas of tissue or background as this confuses the software.

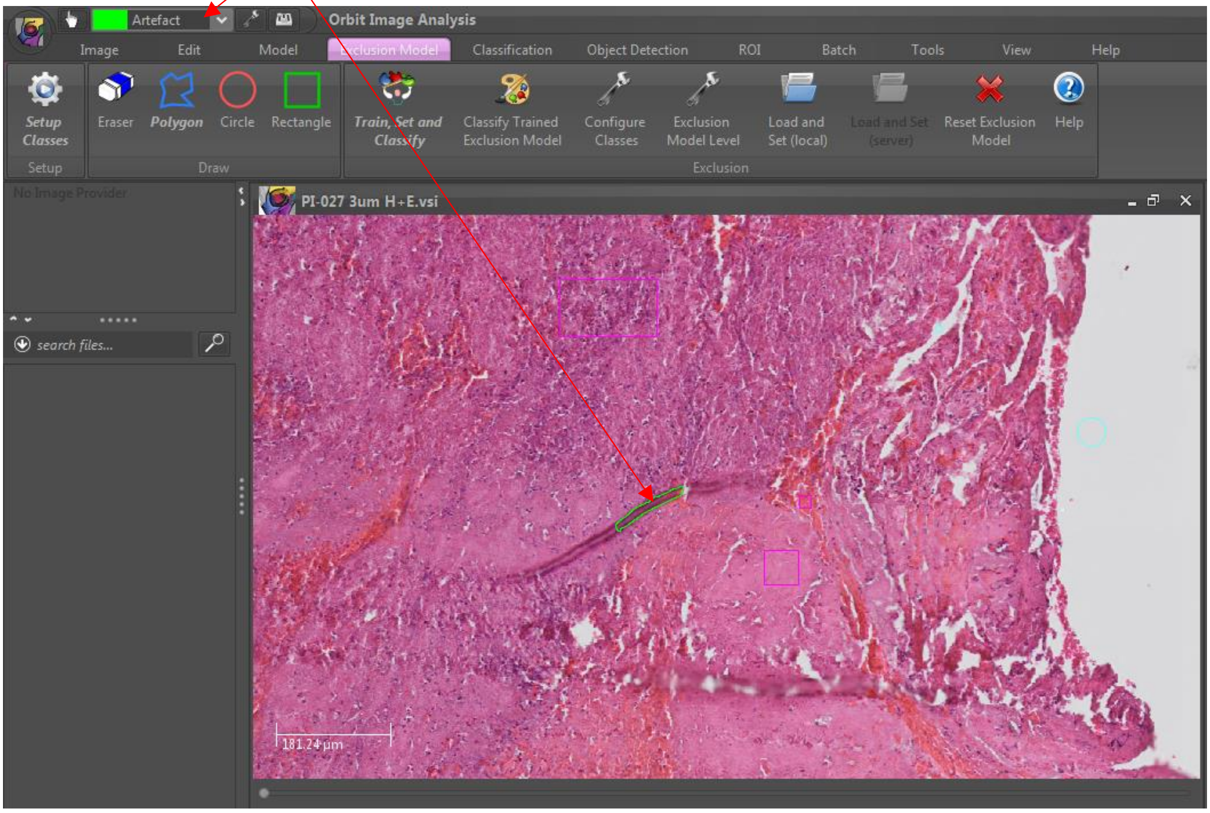

11). On the Exclusion Model tab, Click Train, Set and Classify.

Once it has finished, use the toggle bar at the bottom of the image to see the Exclusion Map.

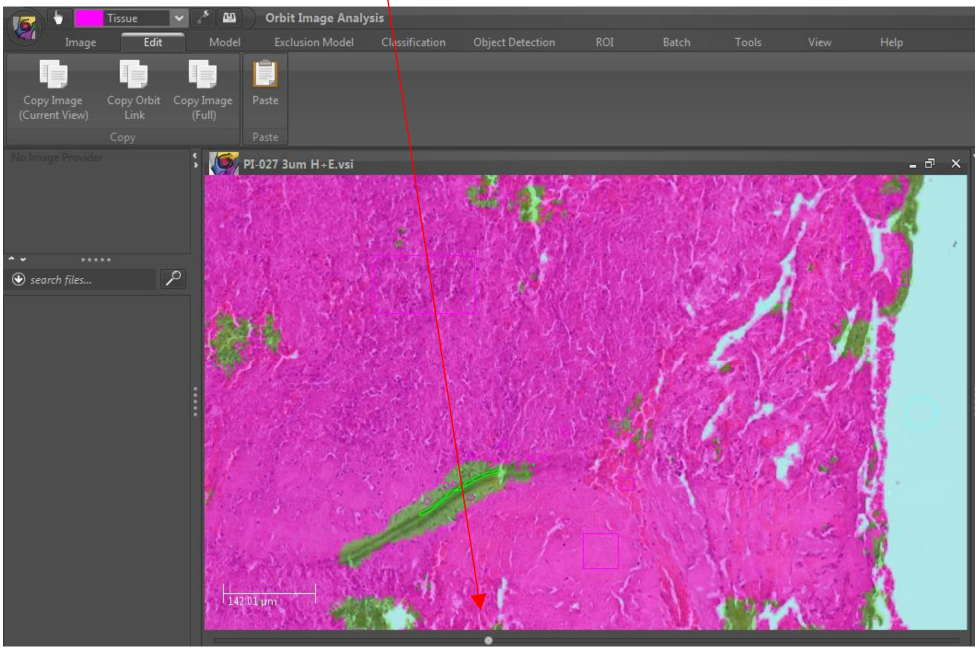

In this case Purple = Tissue (Inclusion), Blue = Background (Exclusion) and Green = Artefact (Exclusion). If you are not happy with your exclusion model, you can repeat steps 8-11 above marking more areas of tissue.

12). If you are happy, click on the Model Tab, then Save model As, select the folder that you wish to save your model in.

***Label this Model as Exclusion Model.**

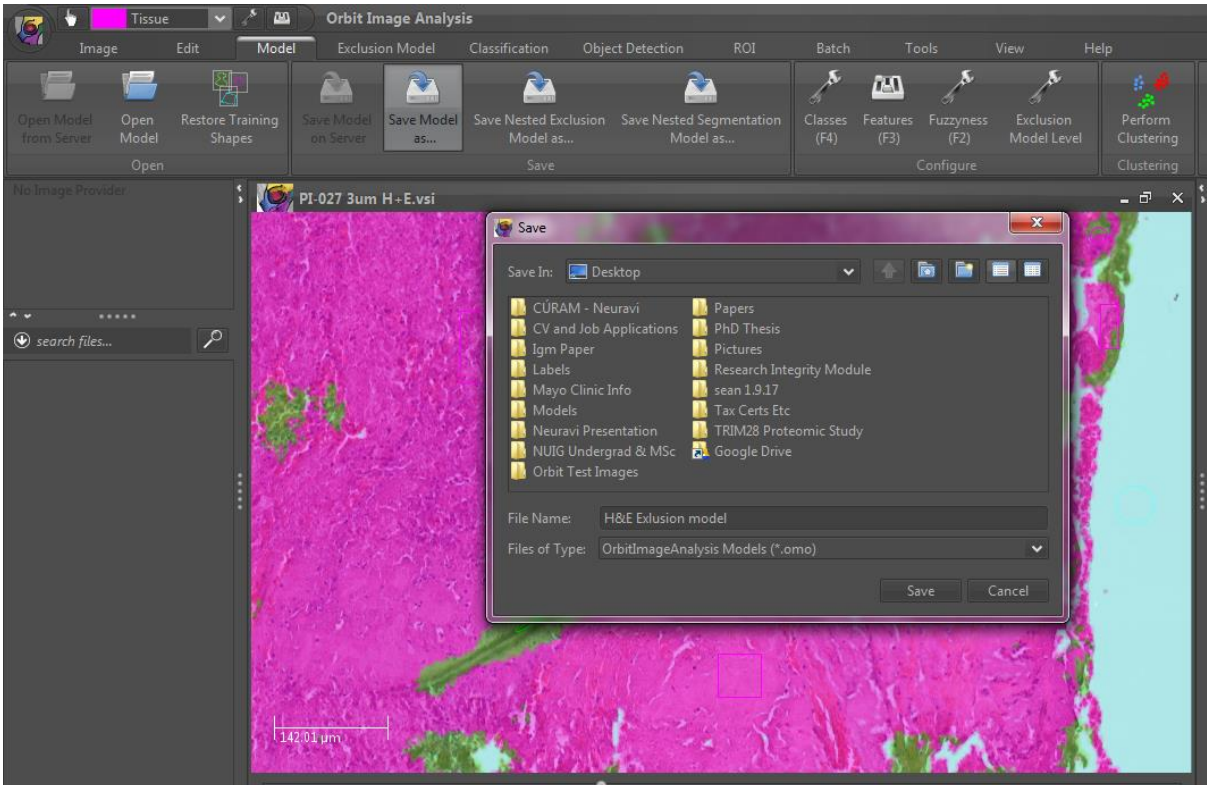

13). **Very Important**: Once the model is saved successfully, click on the Model Tab and then Click Delete Entire Model.

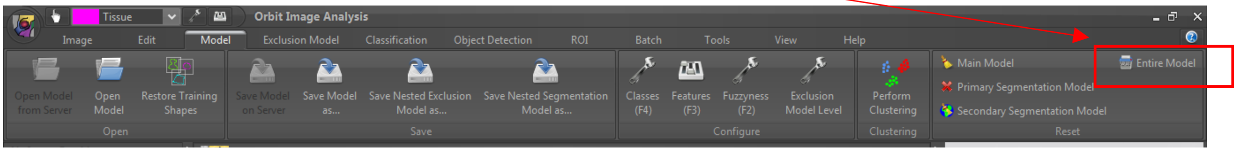

This will reset your classes in the setup classes (Spanner Icon) tab.

14**). Inclusion Model:** Click on the Spanner Icon again and rename the Classes as you wish. You can also change colours and can add or remove classes if necessary.

*All of these classes should be left as undefined. You only define the classes as Inclusion or Exclusion for the Exclusion Model. Click ok.

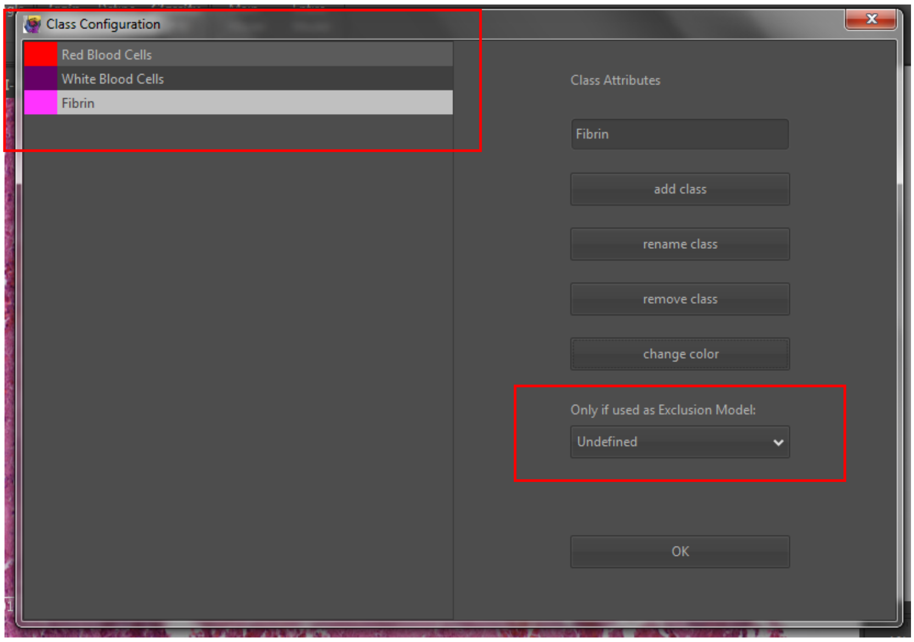

15). Click on the Classification Tab, Select Red Blood Cells and mark areas of Red Blood Cells using the circle, polygon or rectangle tools.

Repeat this for White Blood Cells and Fibrin. (Or whatever your cell types are).

*Make sure that you mark the areas carefully and don't overlap as this will confuse the system. It is important to navigate around the tissue and select cells in different areas as the colours can change slightly throughout the slide.

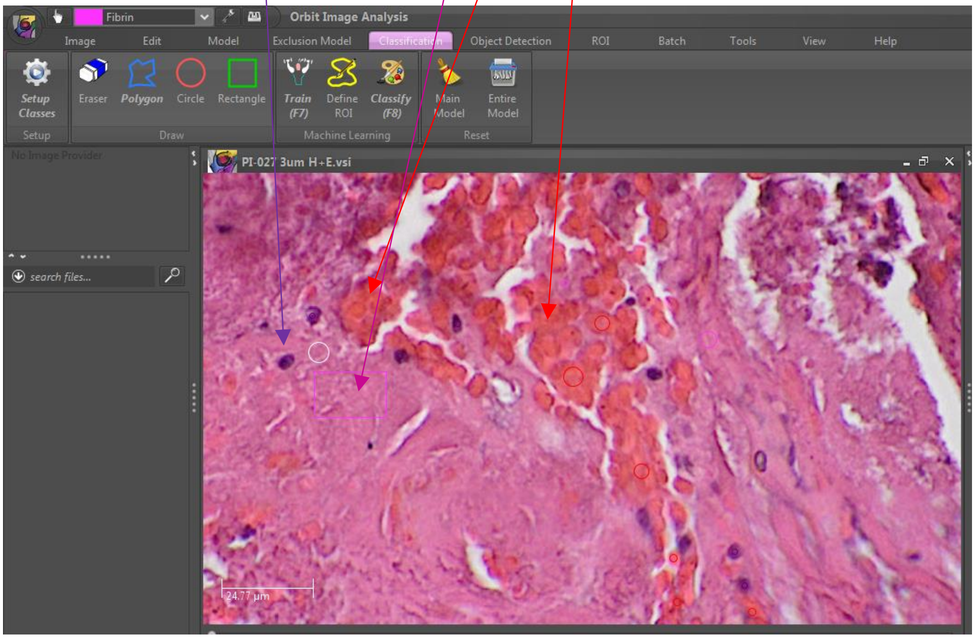

16). On the Classification tab, Click train. Then Click Save Model As, and Save it as Inclusion model.

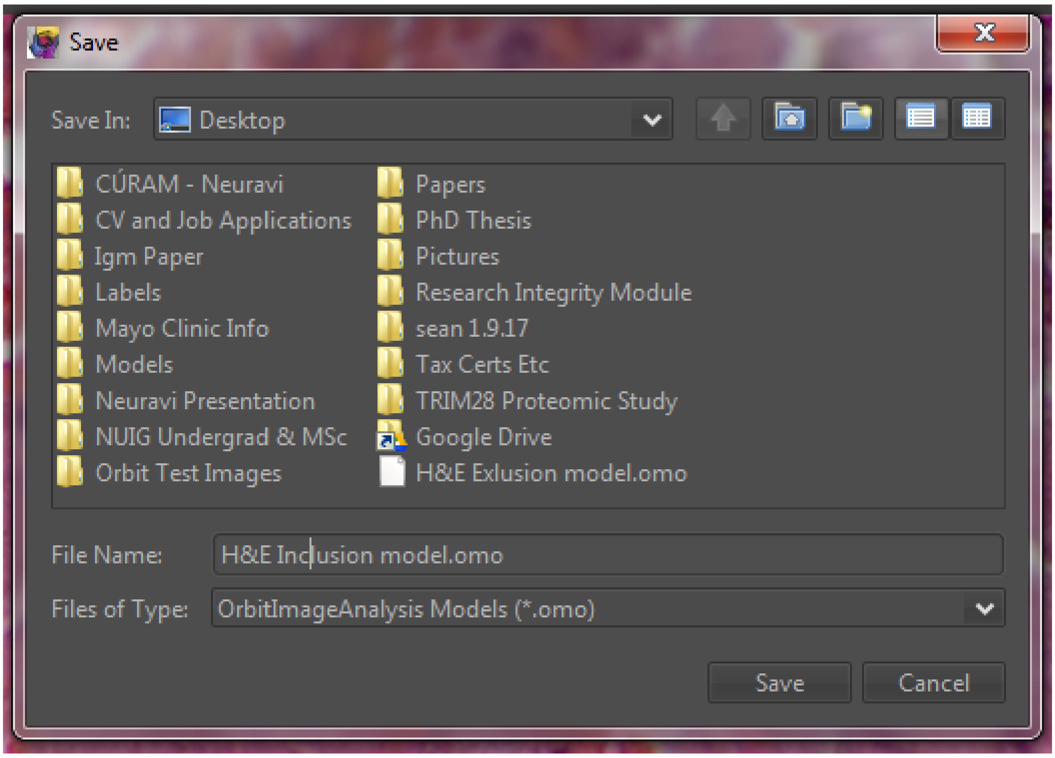

17). Next you need to Apply the Exclusion model that you trained earlier. Under the Exclusion Model Tab, Click on Load and Set (local). Find the exclusion model that you trained earlier and apply it.

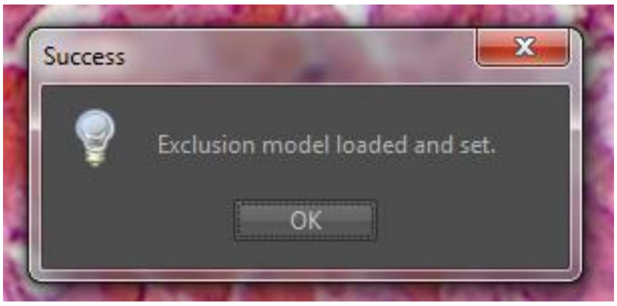

18). Navigate to the Classification Tab and Click on Classify.

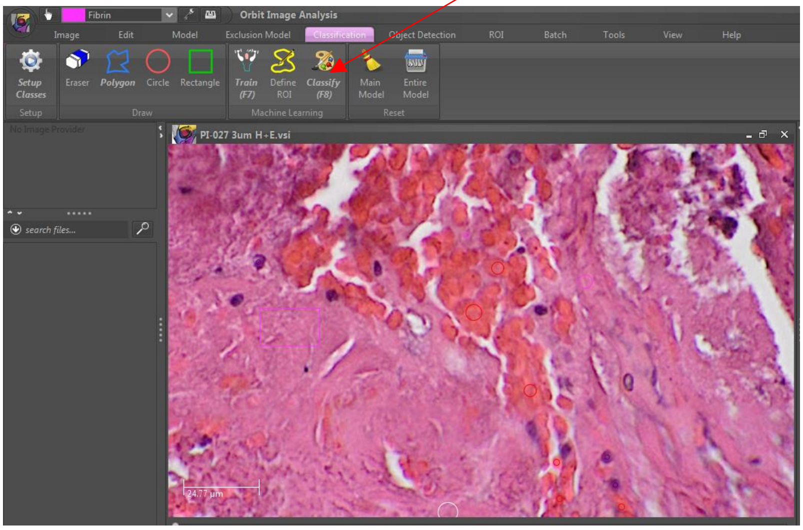

By minimizing the colour adjustment options, you can see the progress of the classification.

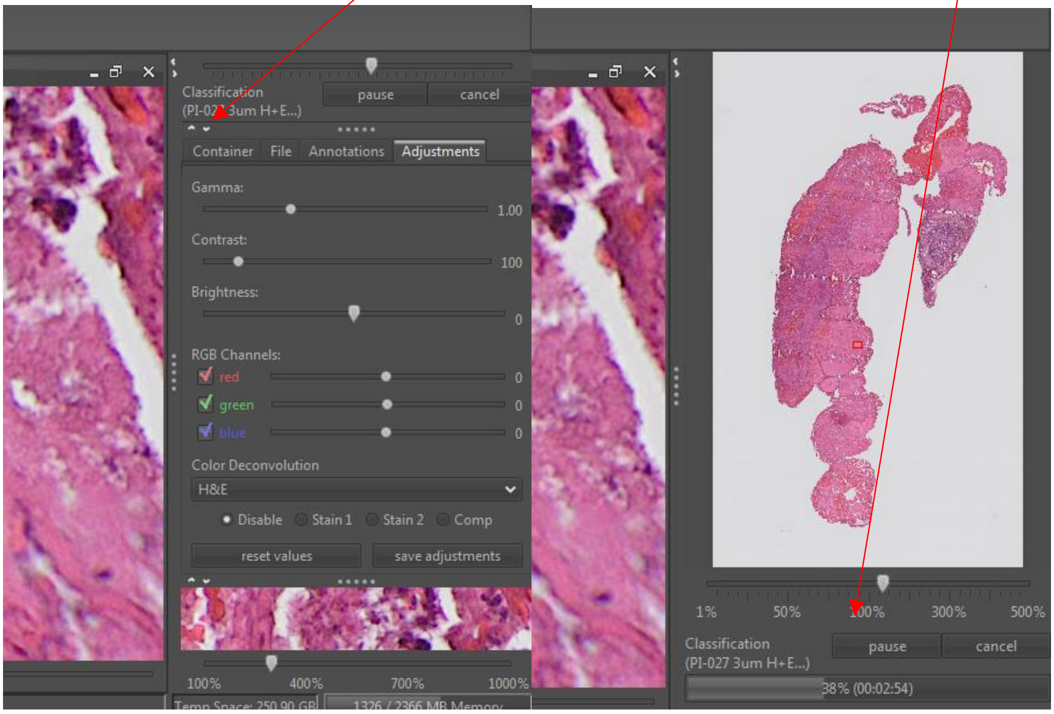

19). Once it is finished classifying, then output will look like this.

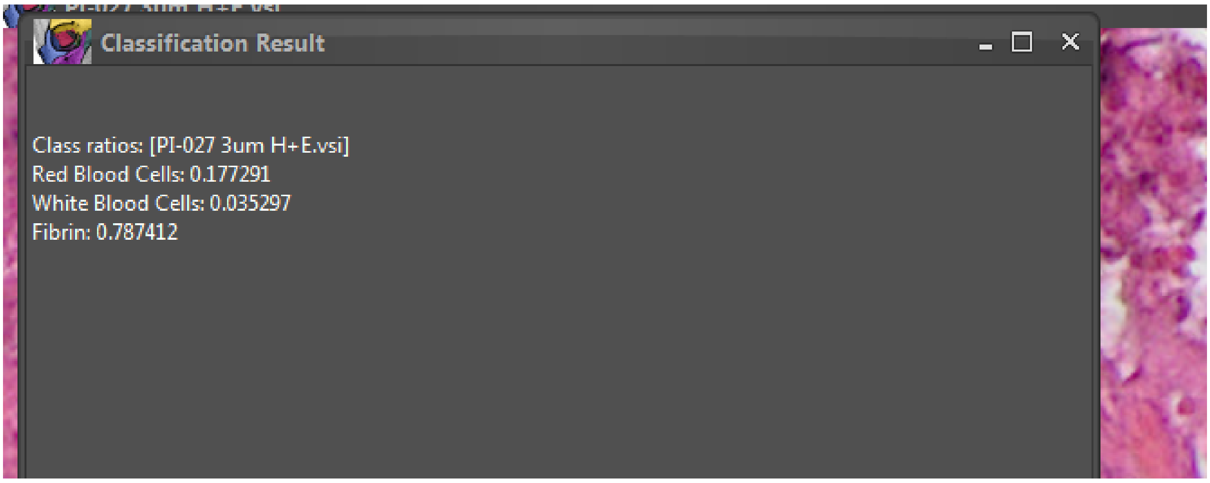

By multiplying each number by 100 you can convert it to a percentage.

You can use the tooglebar at the bottom of the screen to assess how accurate your model was. Red = Red Blood Cells, Purple = White Blood Cells, Pink = Fibrin, White = Background and Grey = Artefact (both Background and Artefact are excluded from the quantification.)

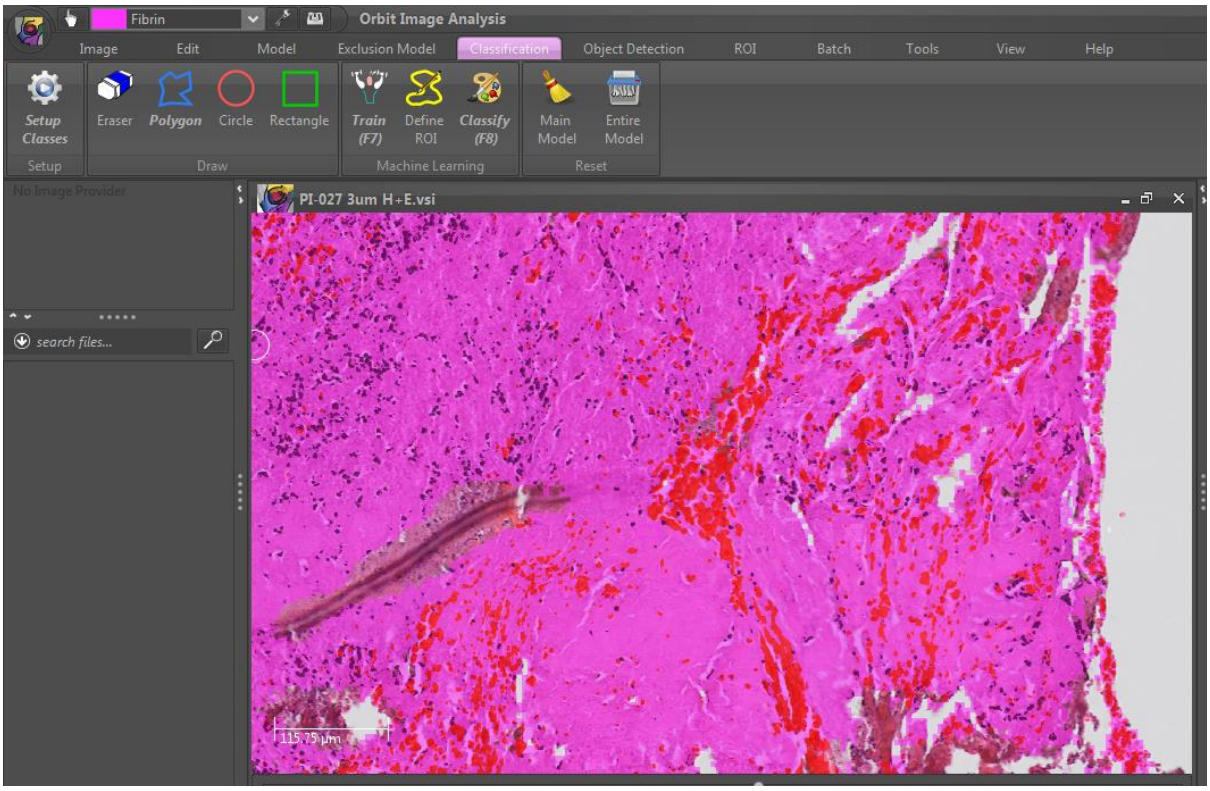

20). If you are happy with your Model, Click Model, Save Model As, and Save it as Main Model. If you are not happy with the Inclusion model, you can repeat steps 15-19, marking more areas of tissue.

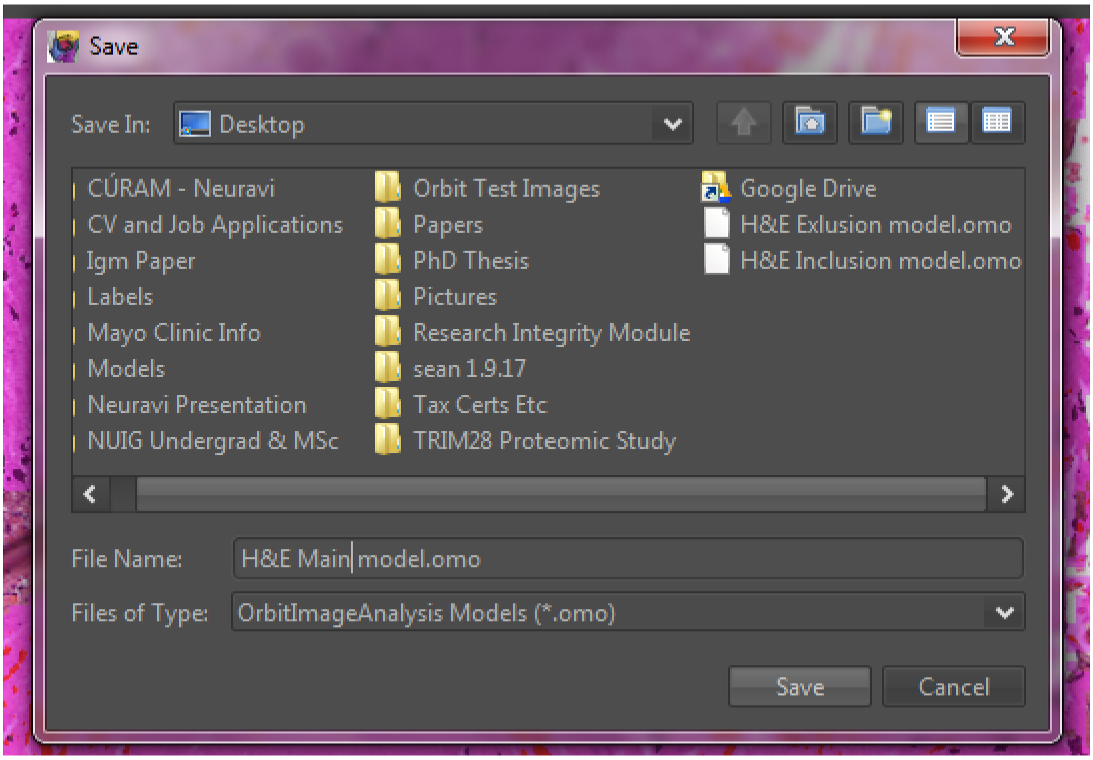

21). Now that you have this model saved and can apply this Main Model to other Slides. When you start the Orbit programme, Click on the model Tab, then open model and open your main model. This Main model includes both your exclusion and inclusion models. Under the classification tab, mark areas of each cell type on the new slide/image and click classify.

Other Important things to note:

- Under the Classification tab you can use the ROI tool to mark regions of interest that you want to analyse instead of analysing the whole slide/image.

- If your Main Model is working well, you can analysing multiple images using Batch mode. First open your main model, then Click on the Batch tab, then Local Area Execution and then select the files that you wish to process.
